# Effect of Postoperative Adverse Events on Hospitalization Expenditures and Length of Stay Among Surgery Patients in Taiwan: A Nationwide Population-Based Case-Control Study

**DOI:** 10.3389/fmed.2021.599843

**Published:** 2021-02-10

**Authors:** Chih-Chieh Yang, Yi-Fei Chuang, Pei-En Chen, Ping Tao, Tao-Hsin Tung, Ching-Wen Chien

**Affiliations:** ^1^Department of Business Administration, Ming Chuan University, Taipei, Taiwan; ^2^Department of Critical Care Medicine, Lotung Poh-Ai Hospital, Yilan, Taiwan; ^3^Institute of Health Policy and Management, National Taiwan University, Taipei, Taiwan; ^4^Taiwan Association of Health Industry Management and Development, Taipei, Taiwan; ^5^Division of Medical Fees, Department of Medical Affairs, Kaohsiung Veteran General Hospital, Kaohsiung, Taiwan; ^6^Enze Medical Research Center, Affiliated Taizhou Hospital of Wenzhou Medical College, Taizhou, China; ^7^Department of Medical Research and Education, Cheng-Hsin General Hospital, Taipei, Taiwan; ^8^Institute for Hospital Management, Tsing Hua University, Shenzhen Campus, Shenzhen, China

**Keywords:** patient safety, patient safety indicators, adverse events patient safety, hospitalization expenditures, length of stay

## Abstract

**Background:** The current study sought to determine the incidence of postoperative adverse events (AEs) based on data from the 2006 Taiwan National Health Insurance Research Database (NHIRD).

**Methods:** This retrospective case-control study included patients who experienced postoperative AEs in 387 hospitals throughout Taiwan in 2006. The independent variable was the presence or absence of 10 possible postoperative AEs, as identified by patient safety indicators (PSIs).

**Results:** A total of 17,517 postoperative AEs were identified during the study year. PSI incidence ranged from 0.1/1,000 admissions (obstetric trauma-cesarean section) to 132.6/1,000 admissions (obstetric trauma with instrument). Length of stay (LOS) associated with postoperative AEs ranged from 0.10 days (obstetric trauma with instrument) to 14.06 days (postoperative respiratory failure). Total hospitalization expenditures (THEs) ranged from 363.7 New Taiwan Dollars (obstetric trauma without instrument) to 263,732 NTD (postoperative respiratory failure). Compared to patients without AEs, we determined that the THEs were 2.13 times in cases of postoperative AE and LOS was 1.72 times higher.

**Conclusions:** AEs that occur during hospitalization have a major impact on THEs and LOS.

## Background

### Adverse Events

Patient safety (PS) is a major healthcare issue worldwide. Previous studies have used the term “adverse events (AEs)” to describe harm to the patient, such as medical injury or iatrogenic injury (1). A report by the Committee on the Quality of Health Care in America (under the Institute of Medicine; IOM), reported that between 44,000 and 98,000 Americans die each year due to medical mistakes, with a corresponding cost of $17–$29 billion (2). Identifying and preventing AEs is a crucial step in dealing with PS.

Previous studies have linked the occurrence of AEs to variables at the patient level and/or hospital level (3). For example, researchers have determined that the risk of AE is proportional to the age of the patient, the number of complications, and the length of stay (LOS) (3–5). The risk of AE is also determined by the type, the size, and the service volume of the hospital (or surgeons), as well as the proportion of beds in the intensive care unit (3, 5). Moonesinghe et al. reported that cases of postoperative AEs are linked to a higher incidence of complications and/or mortality (6). One study reported that the incidence of AEs ranged from 3.7 to 45.8% of the total number of cases (7). Other studies reported that LOS and total hospitalization expenditures (THEs) are far higher in cases involving AE than in cases without incident (3, 4, 8–10).

### Patient Safety Indicators and Adverse Events

AEs are generally identified using reports from care providers or using proxy variables, such as trigger events or other patient safety indicators (PSIs). The preparation of provider-reported events is routine in most medical facilities. Reports of patient-related safety events (e.g., medical errors and near misses) from nurses, physicians, therapists, and other employees are entered into a central database. Harm assessment is based on the status of the patient reported at the time of the event. Trigger events are sentinel words or conditions identified during the initial review of medical records which facilitate the detection of AEs (11). The term PSI refers to a set of computer algorithms developed by the Agency for Healthcare Research and Quality (AHRQ) to identify potential AEs using secondary diagnosis codes from hospital discharge abstracts (12). Note that provider-reported events and trigger events have many technical and administrative constraints, which makes PSIs a more convenient approach to obtaining data of reasonable reliability and validity (11).

### Research Objectives

One previous study reported that the LOS in cases with AEs was 2–3-fold longer than in cases without AEs. They also reported that the in cases with AEs were 2–8-fold higher than in cases without AEs (5). From a clinical standpoint, it is essential to be cognizant of the background information of AEs as well as the complete spectrum of AEs corresponding to THE and LOS. PSIs have proven effective in studying AEs; however, relatively few studies using these methods have been conducted in Asian countries. In the current study, we sought to identify the PSIs for AEs in Taiwan. Specifically, we used the Taiwan National Health Insurance Research Database (NHIRD) to assess the incidence of postoperative adverse events (AEs) in the study population. We also compared cases (with AEs) against matched controls (without AEs) in terms of THE and LOS.

## Methods

### Data Sources

This study analyzed NHIRD inpatient claim datasets from 2006 provided by the Taiwan National Health Research Institute (NHRI). The datasets contain all inpatient claims data, including provider information (contracted medical facilities, board-certified specialists, and medical personnel), enrollee characteristics (age and gender), reasons for using the service (five ICD-9-CM diagnostic and five procedural codes), and financial charges for more than 22 million enrollees covering 98% of the Taiwanese population. The accuracy of the NHIRD has been frequently audited by the Bureau of the National Health Institute (BNHI). Fines for fraud may be as much as 100 times the amount of a false claim charged to the NHI. All personal identifiers are encrypted by the BNHI prior to being released to the public for research purposes; therefore, this study was exempt from a full review by the Institutional Review Board.

### Study Variables

The surgical procedures addressed in this study included those involved in obstetrics & gynecology, orthopedics, neurosurgery, urology, otolaryngology, oral and maxillofacial, digestive, colon and rectal, cardiovascular, chest, pediatric, and cosmetic. The admission pathways included scheduled and emergency interventions.

The dependent variables in this study were THE and LOS resulting from postoperative AEs. THEs were obtained by summing all of the charges for hospitalization due to surgery. LOS was obtained by subtracting the date of discharge from the date of admission for the surgery, which is the same as that used to determine THEs. The independent variable was having one or more of the 10 postoperative AEs vs. none (identified by PSIs; see Appendix). Control variables included the gender, age, and Charlson Comorbidity Index (CCI) score of the patient and hospital size categorized as academic medical centers (AMCs; ≥ 500 acute-care beds), regional hospitals (RHs; ≥ 250 acute-care beds), and district hospitals (DHs; ≤ 250 acute-care beds).

### Statistical Analysis

Statistical analysis was conducted using SAS 9.2 (SAS Institute Inc., Cary, NC, USA). Bivariate analysis was used to assess the associations between independent and dependent variables. Patient age, gender, CCI score, and hospital size were controlled to assess the effects of postoperative AEs on THEs and LOS. THEs were not normally distributed; therefore, logarithmic transformation was performed prior to multiple regression analysis. Poisson regression was used for multivariate analysis due to the fact that excess LOS was a discrete variable, which fit a Poisson distribution when used as the dependent variable in regression analysis. In this study, the level of significance was set at *p* < 0.05.

## Results

### Study Subjects

This retrospective case-control study identified 17,517 postoperative AEs in 2006 from a total of 763,934 cases. We randomly selected 35,034 cases without postoperative AEs to form q control cohort at a ratio of 1:2 (13). Note that patients were matched for age (±1 year), gender, and the first three diagnosis codes (ICD-9-CM) related to surgery as well as the size of the hospital in which the surgery was performed.

As shown in Table 1, no differences were observed in terms of patient age, gender, or the size of the hospital. However, the CCI scores of subjects who experienced AE were significantly higher than those who did not experience AE (1.32 ± 1.88 vs. 1.05 ± 1.82, respectively; *p* < 0.001).

**Table 1:** Association between control variables and the occurrence of postoperative adverse event (AEs).

	**Suffering from postoperative AEs or not**			
	**Total**	**Yes**	**No**	***p*-value**
	***N* (%)**			
Total	52,551 (100)	17,517 (100)	35,034 (100)	
Gender				>0.99^a^
Male	24,027 (45.7)	8,009 (45.7)	16,018 (45.7)	
Female	28,524 (54.3)	9,508 (54.3)	19,016 (54.3)	
Hospital size				>0.99^a^
District hospitals	7,653 (14.6)	2,551 (14.6)	5,102 (14.6)	
Regional hospitals	23,400 (44.5)	7,800 (44.5)	15,600 (44.5)	
Academic medical centers	21,498 (40.9)	7,166 (40.9)	14,332 (40.9)	
**Mean (SD)**				
Age	56.12 (20.91)	56.31 (21.10)	56.02 (20.82)	0.14^b^
Charlson Comorbidity Index (CCI) score	1.14 (1.85)	1.32 (1.88)	1.05 (1.82)	<0.001^b^

a *chi-square test*;

b *t-test*.

Table 2 lists the average number of postoperative AEs (PSIs) at the 387 hospitals included in the current study, which accounted for 78% of all hospitals in Taiwan in 2006. The average number of postoperative AEs was 47.12 per hospital in 2006, which accounted for 2.4% of all inpatient surgeries. Postoperative AEs included the following: obstetric trauma with instrument (10,129,765), obstetric trauma without instrument (2,864,753), postoperative respiratory failure (962,557), postoperative sepsis (458,360), postoperative physiologic and metabolic derangement (175,705), postoperative pulmonary embolism or deep venous thrombosis (84,033), postoperative hemorrhage or hematoma (68,754), postoperative hip fracture (30,557), and obstetric trauma-cesarean section (7,639). The three most frequent postoperative AEs were postoperative respiratory failure (22.00), postoperative sepsis (8.48) and obstetric trauma without instrument (7.35). The three most frequent postoperative AEs in terms of incidence were obstetric trauma with instrument (13.3%), obstetric trauma without instrument (3.8%), and postoperative respiratory failure (1.3%). As shown in Table 3, postoperative AEs varied considerably in terms of total hospitalization expenditures (THEs) and length of stay (LOS). As shown in Table 4, the THEs and LOS associated with cases involving AE exceeded those associated with cases without incident. The excess THEs due to each AE was NTD 162,422 (New Taiwan Dollars; one USD = 32.71 NTD in 2006) and the excess LOS was 9.21 days. Only the excess LOS for obstetric trauma without instrument and obstetric trauma-cesarean section did not reach the level of statistical significance. The three PSIs with the highest excess THEs were postoperative respiratory failure (NTD 263,732), postoperative sepsis (NTD 169,644) and postoperative physiologic and metabolic derangement (NTD 138,242). The three PSIs with the highest excess LOS were postoperative respiratory failure (14.06 days), postoperative sepsis (9.84 days) and postoperative hip fracture (9.42 days).

**Table 2:** Description of the postoperative adverse event (AEs)-Patient safety indicators (PSIs) that occurred at 387 Taiwan hospitals in 2006 (n = 763,934).

**PSIs**	**Frequencies**	**Mean of frequencies (Incidence %)**
Postoperative hip fracture	30,557	0.51 (0.04)
Postoperative hemorrhage or hematoma	68,754	1.55 (0.1)
Postoperative physiologic and metabolic derangement	175,705	4.02 (0.2)
Postoperative respiratory failure	962,557	22.00 (1.3)
Postoperative pulmonary embolism or deep venous thrombosis	84,033	2.00 (0.1)
Postoperative sepsis	458,360	8.48 (0.6)
Obstetric trauma-vaginal with instrument	10,129,765	4.10 (13.3)
Obstetric trauma-vaginal without instrument	2,864,753	7.35 (3.8)
Obstetric trauma-cesarean section	7,639	0.02 (0.01)
The number of postoperative AEs		47.12 (2.4)

**Table 3:** The trend of medical resource consumption owing to adverse event (AEs).

	**Adverse event (AEs)**		
	**No (*n* = 35,034)**	**Yes (*n* = 17,517)**	***p*-value for *t*-test**
	**Mean (SD)**		
Total hospitalization expenditures (THEs)	114,607.42 (159,220.99)	276,902.77 (306,837.65)	<0.001
Length of stay (LOS)	11.89 (13.39)	21.00 (19.55)	<0.001

**Table 4:** Univariate analysis of the postoperative PSIs based on medical resource consumption (n = 52,551).

	**Total hospitalization expenditures (THEs)**^****a****^		**Length of stay (LOS)**^****a****^	
	**Excess THEs**	***p*-value**	**Excess LOS**	***p*-value**
**Postoperative adverse event (AEs)**				
Yes (Reference group: No)	162,422	<0.001	9.21	<0.001
Postoperative hip fracture		<0.001		
Yes (Reference group: No)	75,356.6	<0.001	9.42	<0.001
Postoperative hemorrhage/hematoma		<0.001		<0.001
Yes (Reference group: No)	111,383	<0.001	6.37	<0.001
Postoperative physiologic and metabolic derangement		<0.001		
Yes (Reference group: No)	138,242	<0.001	7.00	<0.001
Postoperative respiratory failure		<0.001		<0.001
Yes (Reference group: No)	263,732	<0.001	14.06	<0.001
**Postoperative pulmonary embolism or deep venous thrombosis**				
Yes (Reference group: No)	33,559.8	0.0002	5.41	<0.001
Postoperative sepsis				<0.001
Yes (Reference group: No)	169,644	<0.001	9.84	<0.001
Obstetric trauma-vaginal with instrument		<0.001		<0.001
Yes (Reference group: No)	560.3	<0.001	0.10	<0.001
Obstetric trauma-vaginal without instrument		<0.001		
Yes (Reference group: No)	363.7	<0.001	0.02	0.17
Obstetric trauma-cesarean section				
Yes (Reference group: No)	6,399.0	0.03	0.67	0.13

a *t-test*.

Multiple regression was used to assess the effects of independent risk factors on THEs and LOS. Table 5 lists the THEs and LOS where age, gender, hospital size, and CCI score are included in the model as control variables. The THEs associated with cases involving postoperative AEs were 2.13-fold (95% CI: 2.09–2.17) higher than those without AEs. The LOS associated with postoperative AEs was 1.72-fold (95% CI: 1.69–1.75) higher. Both of these effects reached the level of significance. The control variables also presented significant effects. THEs and LOS were both positively correlated with age and CCI score as well as sex (male) and hospital size (AMCs > RHs > DHs).

**Table 5:** Analysis of the occurrence of adverse event (AEs), other variables, and medical consumption.

	Total hospitalization expenditures (THEs)^*^^,^^a^			**Length of stay (LOS)**^**b**^		
	**Hazard Ratio**	**95%CI**	***p*-value**	**Hazard Ratio**	**95%CI**	***p-value***
**Suffering from postoperative AEs or not**						
Yes (Reference group: No)	2.13	2.09–2.17	<0.001	1.72	1.69–1.75	<0.001
**Gender**						
Male (Reference group: Female)	1.52	1.49–1.55	<0.001	1.31	1.28–1.34	<0.001
**Hospital size**						
Academic medical centers (Reference group: regional hospitals)	1.21	1.19–1.23	<0.001	1.09	1.07–1.11	<0.001
District hospitals	0.73	0.72–0.74	<0.001	0.86	0.84–0.88	<0.001
**Age**	1.02	1.00–1.04	<0.001	1.02	1.00–1.04	<0.001
Charlson Comorbidity Index (CCI) score	1.12	1.09–1.14	<0.001	1.08	1.05–1.10	<0.001

a *Multiple regression*;

b *Poisson regression*;

* *Logarithmic transformation*.

Table 6 presents the different AEs for patients who underwent surgeries in various populations (14–20).

**Table 6:** The comparison among other national audits.

**References**	**Screened number**	**Following period**	**Setting**	**Mortality OR (95% CI)**	**Adverse events (AEs)**	**THEs or LOS**
Rosen et al. (14)	8,126 (CABG = 2,213 PTCA = 693 Cholecystectomy = 2,863 Prostatectomy = 2,357)	1985, Jan.- 1986, Jun	USA	*30-day mortality rates ranged from 1 0 to 6.6%* CABG patients with AEs had higher mortality rates (15.2 vs. 2.6%, *P* < 0.001)	*The prevalence of postoperative adverse events identified from chart review was greater (6.9–33.3%) *The R-square values using clinical indicators at admission to predict the occurrence of any adverse event ranged from 0.05 to 0.13.	*LOS, days (Yes AEs vs. No AEs)* CABG 15.8 ± 10.4 vs. 9.2 ± 5.2 (*p* < 0.001) PTCA 11.4 ± 8.7 vs. 6.7 ± 5.0 (*p* < 0.001) Cholecystectomy 15.8 ± 10.4 vs. 9.2 ± 5.2 (*p* < 0.001) Prostatectomy 11.1 ± 7.0 vs. 6.3 ± 3.9 (*p* < 0.001)
Seglenieks et al. (15)	1,291 surgical patients	2012/02/07−2012/03/16	South Australia	In postoperative wards, mortality was low in the first 48 h postoperatively	AEs in the postoperative care unit were common, including desaturation (13.6%), hypotension (5.8%) and apnoea (5.5%), with 19.9% of cases requiring attendance by an anesthetist to manage unexpected complications	Incidence of adverse events in the post-anesthesia care unit (PACU) after the first 30 minLOS, days: ≥ 2 h:442 (35.8%) LOS, days: ≥ 4 h:103 (8.3%)
Lin et al. (16)	24,109 surgical patients with preoperative asthma and 24,109 non-asthma patients undergoing major surgeries using matching procedure with propensity score	2008–2010	Taiwan	Preoperative emergency care for asthma was significantly associated with postoperative 30-day in-hospital mortality, 1.84 (1.11–3.04)	Asthma increased postoperative pneumonia (OR 1.48; 95% CI 1.34–1.64), septicemia (OR 1.11; 95% CI 1.02–1.21), and urinary tract infection (OR 1.17; 95% CI 1.09–1.26)	NA
Yeh et al. (17)	7,973 physicians as surgical patients and 7,973 propensity score–matched non-physician controls receiving in-hospital major surgeries	2004–2010	Taiwan	Physicians as surgical patients were not associated with 30-day in-hospital mortality after surgery	Physicians as surgical patients had lower adjusted ORs with 95% CIs of postoperative deep wound infection (OR 0.63, 95%CI 0.40–0.99; *P* < 0.05), ICU admission (OR 0.74, 95% CI 0.66–0.83; *P* < 0.0001)	Physicians vs. Non-physicians Increased medical expenditure, USD: OR 0.80, 95% CI 0.73–0.88; *P* < 0.0001). prolonged LOS, days: (OR 0.68, 95% CI 0.62–0.75, *P* < 0.0001)
Lin et al. (18)	16,539 diabetes patients(aged >20 years, who underwent major surgery) 16,539 propensity score–matched non-diabetes controls	2009–2015	Taiwan	In-hospital mortality 1.51 (1.07–2.13) compared to people without diabetes	Patients with diabetes had a higher risk of postoperative septicemia (OR 1.33, 95% CI 1.01–1.74), necrotizing fasciitis (OR 3.98, 95% CI 1.12–14.2), cellulitis (OR 2.10, 95% CI 1.46–3.03), acute pyelonephritis (OR 1.86, 95% CI 1.01–3.41) and infectious arthritis (OR 3.89, 95% CI 1.19–12.7), compared to people without diabetes. Previous admission for diabetes (OR 2.33, 95% CI 1.85–2.93), HbA1c >8% (OR 1.96, 95% CI 1.64–2.33) and fasting glucose >180 mg/dL (OR 1.90, 95% CI 1.68–2.16) were predictors for post-operative adverse events	DM vs. Non-DM Medical expenditure, USD: 5,167 ± 5,720 vs. 4,221 ± 4,693 (*p* < 0.0001) LOS, days: 9.1 ± 14.5 vs. 7.1 ± 10.1 (*p* < 0.0001)
Wang et al. (19)	1,525 patients	2010, Jan.- 2015, Oct	China	death (*n* = 3, 2.5%)	Multivariable regression analysis showed that age >65 years old (OR 2.23, 95%CI: 1.25–3.98), male sex (OR = 2.14, 95% CI: 1.24–3.38), postoperative peripheral blood glucose (OR = 1.13, 95% CI: 1.13–1.82), diabetic complications (OR = 2.41, 95% CI: 1.36–4.28), abnormal kidney function (OR = 2.73, 95% CI: 1.13–6.58) and general surgery (OR = 1.48, 95% CI: 1.11–5.26) were associated with the occurrence of postoperative AEs	*Overall = 14.65 ± 12.06*With AEs vs. No AEs27.82 ± 26.96 vs. 13.43 ± 8.53 (*p* = 0.0002)
Cherng et al. (20)	35,643 patients with CKD who underwent non-urological surgeries. 35,643 propensity score–matched non-CKD controls	2008–2013	Taiwan	CKD vs. Non-CKD in-hospital mortality 2.17 (1.90–2.47)	Patients with CKD had higher risks of postoperative septicemia (OR: 1.78, 95% CI: 1.68–1.89), pneumonia (OR: 1.60, 95% CI: 1.48–1.73), stroke (OR: 1.34, 95% CI: 1.24–1.44), and in-hospital mortality (OR: 2.17, 95% CI: 1.90–2.47) compared with non-CKD patients	Medical expenditure, USD (CKD vs. Non-CKD): 4,598 ± 5,700 vs. 3,938 ± 5,195 (*p* < 0.0001)LOS, days (CKD vs. Non-CKD): 13.2 ± 17.8 vs. 11.0 ± 16.5 (*p* < 0.0001)

## Discussion

### Clinical Implications

This study used PSIs to identify the occurrence of postoperative AEs in the Taiwanese healthcare system, and then examined the effects of these events on resource consumption (THEs and LOS). Regression analysis revealed that the AEs identified in this study led to substantial increases in the duration of hospital stays and medical expenditures. Postoperative sepsis, postoperative physiologic and metabolic derangement, and postoperative hip fracture had the most profound consequences.

Among the postoperative AEs that occurred in 2006, those with the highest incidence were obstetric trauma with instrument (13.26%) and obstetric trauma without instrument (3.75%), which is similar to findings in other countries. Zhan et al. identified obstetric trauma with instrument as the most frequent postoperative PSI (22.42%), followed by obstetric trauma without instrument (8.66%) (9). Bottle et al. identified postoperative PSI obstetric trauma with instrument as the most frequent PSI (6.05%), followed by obstetric trauma without instrument (2.79%) (4). Note that they discussed only 9 PSIs in that study. Raleigh et al. identified obstetric trauma with instrument as the most frequent PSI (5.39%), followed by obstetric trauma without instrument (2.61%) (10). In the current study, the annual incidence of postoperative wound dehiscence was zero, which is in line with the results of a previous study conducted in Taiwan. The authors of the earlier study speculated that this could be attributed to difficulties in coding postoperative wound dehiscence (21).

We also investigated the effects of postoperative AEs on THEs and LOS, after correcting for patient variables (gender, age, and CCI score) and hospital characteristics (hospital size). Overall the THEs and LOS in cases involving postoperative AEs were significantly higher than those in cases without AEs, which is in agreement with previous studies (3, 4, 8–10). Postoperative respiratory failure had the most pronounced effect on THEs and LOS” and “Other events with a profound effect on THEs and LOS included the following: postoperative sepsis (NTD 169,644; 9.84 days), postoperative physiologic and metabolic derangement (NTD 138,242; 7.00 days), postoperative hemorrhage/hematoma (NTD 111,383; 6.37 days), and postoperative hip fracture (NTD 75,357; 9.42 days). Our findings pertaining to hospital stays are in agreement with the results obtained in earlier studies: postoperative respiratory failure [8.6–17 days (3, 8, 9)], postoperative sepsis [10.89–18.8 days (3, 4, 8–10)], postoperative physiologic and metabolic derangement [8.89–15 days (8, 9)], postoperative hemorrhage/hematoma [3.9–6 days (3, 8, 9)], and postoperative hip fractures [0.24–17.09 days (4, 8–10)]. Our findings pertaining to hospital costs are also in good agreement with the results obtained in earlier studies: postoperative respiratory failure [USD 28,218–53,502 (3, 8, 9, 22)], postoperative sepsis [USD 31,264–57,727 (3, 8, 9)], postoperative physiologic, and metabolic derangement [USD 37,460–54,818 (8, 9)], postoperative hemorrhage/hematoma [USD 7,863–21,431 (3, 8, 9)], and postoperative hip fractures [USD 13,441–18,906 (8, 9)]. In exploring the impact of AEs on 90-day medical expenditures, Encinosa and Hellinger found that AEs increased THEs by an average of 20%. Among these AEs, acute respiratory failure resulted in the greatest increase (52%), followed by infection caused by medical care and postoperative sepsis (42%), postoperative hip fracture and pressure ulcers (32.9%), and postoperative physiologic and metabolic derangement (31.8%). Some events also imposed considerably higher readmission rates and corresponding expenditures, including infection caused by medical care and postoperative sepsis, postoperative physiologic and metabolic derangement, and acute respiratory failure (22).

Our findings demonstrated the efficacy of using PSIs to identify AEs. There is currently a lack of research on PSIs in Asian countries. Thus, the current study could serve as a reference, facilitating comparisons among Asian countries.

As in previous studies, we considered potential differences in sample size associated with case cohort vs. control cohort studies. To reduce individual bias, created a control cohort matched for age, gender, hospital size, and the first three ICD-9 codes at a ratio of 1:2. We also considered both patient and hospital characteristics as control variables to eliminate interference from factors that might otherwise skew our results. From other studies (Table 6), they also showed the patients who underwent surgeries with operative AEs, would have higher THEs also longer LOS.

### Limitation

A number of limitations must be considered in the interpretation of our results. First, we opted for old data to avoid the drastic changes in procedures in recent years. Second, our use of PSIs as a proxy for AEs was not assessed in the present study. Third, the selection of subjects who had undergone postoperative AEs was based on ICD-9 codes in administrative databases; therefore, we cannot rule out the possibility of data entry errors. Fourth, financial incentives or disincentives might have resulted in up-coded related diagnoses on the medical records. Nonetheless, these events are rarely observed in Taiwan due to the strict inpatient diagnosis requirements stipulated by the BNHI. Fifth, the findings in this population-based study should be generalizable to the overall population; however, evidence derived from case-control studies is generally lower in statistical quality than that obtained from randomized trials, due to biases related to adjustments for confounding variables (23). Sixth, despite our meticulous study design and measures to control for confounding factors, bias resulting from unknown confounders may still have affected these results. Finally, all the data in the NHIRD was anonymous; therefore, many relevant clinical variables (e.g., serum laboratory data, imaging results, and pathology findings) were unavailable.

## Conclusions

This study demonstrated that AEs during hospitalization can be identified by PSIs, and that these events can have a profound impact on THEs and LOS. Further studies will be required to elucidate the temporal sequence of AEs that typically lead to elevated THEs and LOS. Research will also be required to explore other factor-related differences that appear to be associated with THEs and LOS.

## Data Availability Statement

The raw data supporting the conclusions of this article will be made available from the corresponding author upon request.

## Author Contributions

C-CY, Y-FC, PT, P-EC, T-HT, and C-WC conducted the study and drafted the manuscript. Y-FC and PT participated in the design of the study and performed statistical analyses. C-CY, T-HT, and C-WC conceived the study and participated in its design and coordination. All authors read and approved the final manuscript.

## Conflict of Interest

The authors declare that the research was conducted in the absence of any commercial or financial relationships that could be construed as a potential conflict of interest.

## Abbreviations

NHIRD: National Health Insurance Research Database
PSIs: Patient Safety Indicators
AEs: Adverse events
THEs: Total hospitalization expenditures
LOS: Length of stay
CCI: Charlson Comorbidity Index
NTD: New Taiwan Dollars
PS: Patient safety
IOM: Institute of Medicine
AHRO: Agency for Healthcare Research and Quality
NHRI: National Health Research Institute
BNHI: Bureau of the National Health Institute
AMCs: Academic medical centers
RHs: Regional hospitals
DHs: District hospitals.
