# Supplementary material for: Effect of Postoperative Adverse Events on Hospitalization Expenditures and Length of Stay Among Surgery Patients in Taiwan: A Nationwide Population-Based Case-Control Study
Source: Front Med (Lausanne). 2021 Feb 10;8:599843. doi: 10.3389/fmed.2021.599843 (PMC7902791; doi:10.3389/fmed.2021.599843)
Supplement: Supplementary file 1 [file Table_1.docx]

**Appendix:** The definition of postoperative PSIs

| **PSI#8 Postoperative Hip Fracture** | |
| --- | --- |
| **Numerator** | Discharges with ICD-9-CM code for hip fracture in any secondary diagnosis field among cases meeting the inclusion and exclusion rules for the denominator. |
| **Denominator** | All surgical discharges age 18 and older defined by specific DRGs or MS-DRGs and an ICD-9-CM code for an operating room procedure.  **Exclude Cases:**   - with principal diagnosis of hip fracture or secondary diagnosis present on admission. - where the only operating room procedure is hip fracture repair. - where a procedure for hip fracture repair occurs before or on the same day as the first operating room procedure. - with diseases and disorders of the musculoskeletal system and connective tissue (MDC 8). - with principal diagnosis (or secondary diagnosis present on admission, if known) of seizure, syncope, stroke, coma, cardiac arrest, poisoning, trauma, delirium and other psychoses, or anoxic brain injury. - with any diagnosis of metastatic cancer, lymphoid malignancy or bone malignancy, or self-inflicted injury. - MDC14 (pregnancy, childbirth, and puerperium). |
| **PSI#9 Postoperative Hemorrhage or Hematoma** | |
| **Numerator** | ICD-9-CM code for postoperative hemorrhage or postoperative hematoma in any secondary diagnosis field **AND** ICD-9-CM code for postoperative control of hemorrhage or for drainage of hematoma procedure code. |
| **Denominator** | All surgical discharges age 18 and older defined by specific DRGs or MS-DRGs and an ICD-9-CM code for an operating room procedure.  **Exclude Cases:**   - with principal diagnosis of postoperative hemorrhage or postoperative hematoma or secondary diagnosis present on admission. - where the only operating room procedure is postoperative control of hemorrhage or drainage of hematoma. - where a procedure for postoperative control of hemorrhage or drainage of hematoma occurs before the first operating room procedure. - MDC 14 (pregnancy, childbirth, and puerperium). |
| **PSI#10 Postoperative Physiologic and Metabolic Derangement** | |
| **Numerator** | Discharges among cases meeting the inclusion and exclusion rules for the denominator with ICD-9-CM codes for physiologic and metabolic derangements in any secondary diagnosis field.  Discharges with acute kidney failure (subgroup of physiologic and metabolic derangements) must be accompanied by a procedure code for dialysis. |
| **Denominator** | All elective* surgical discharges18 years and older defined by specific DRGs or MS-DRGs and an ICD-9-CM code for an operating room procedure.  *Elective - SID Admission type # is recorded as elective (Admission Type = 3)  **Exclude cases:**   - with preexisting condition (principal diagnosis or secondary diagnosis present on admission) of physiologic and metabolic derangements. - with acute renal failure (see Numerator) where a procedure for dialysis occurs before or on the same day as the first operating room procedure. - with both a diagnosis code of ketoacidosis, hyperosmolarity, or other coma (subgroups of physiologic and metabolic derangements coding) and a principal diagnosis of diabetes. - with both a secondary diagnosis code for acute renal failure (subgroup of physiologic and metabolic derangements coding) and a principal diagnosis of acute myocardial infarction, cardiac arrhythmia, cardiac arrest, shock, hemorrhage, or gastrointestinal hemorrhage. - MDC 14 (pregnancy, childbirth and the puerperium). |
| **PSI#11 Postoperative Respiratory Failure** | |
| **Numerator** | Discharges among cases meeting the inclusion and exclusion rules for the denominator. With ICD-9-CM codes for acute respiratory failure in any secondary diagnosis field. **OR** Discharges among cases meeting the inclusion and exclusion rules for the denominator with ICD-9-CM codes as follows:   - Mechanical Ventilation for 96 consecutive hours or more - zero or more days after the major operating room procedure code. - Mechanical Ventilation for less than 96 consecutive hours or undetermined - two or more days after the major operating room procedure code. - Reintubation - one or more days after the major operating room procedure code. |
| **Denominator** | All elective* surgical discharges age 18 and older defined by specific DRGs or MS-DRGs and an ICD-9-CM code for an operating room procedure.  *Elective - SID Admission type # is recorded as elective (Admission Type = 3)  **Exclude cases:**   - with principal diagnosis of acute respiratory failure or secondary diagnosis present on admission. - with ICD-9-CM diagnosis code of neuromuscular disorder. - where a procedure for tracheostomy is the only operating room procedure. - where a procedure for tracheostomy occurs before the first operating room procedure. - with any diagnosis code of craniofacial anomalies with   1) a procedure code for laryngeal or pharyngeal surgery or  2) a procedure on face.   - MDC 14 (pregnancy, childbirth, and puerperium). - MDC 4 (diseases/disorders of respiratory system). - MDC 5 (diseases/disorders of circulatory system). |
| **PSI#12 Postoperative Pulmonary Embolism or Deep Vein Thrombosis** | |
| **Numerator** | Discharges among cases meeting the inclusion and exclusion rules for the denominator. With ICD-9-CM codes for deep vein thrombosis or pulmonary embolism in any secondary diagnosis field. |
| **Denominator** | All surgical discharges age 18 and older defined by specific DRGs or MS-DRGs and an ICD-9-CM code for an operating room procedure.  **Exclude cases:**   - with principal diagnosis of deep vein thrombosis or pulmonary embolism or secondary diagnosis present on admission. - where a procedure for interruption of vena cava is the only operating room procedure. - where a procedure for interruption of vena cava occurs before or on the same day as the first operating room procedure. - MDC 14 (pregnancy, childbirth, and puerperium). |
| **PSI#13 Postoperative Sepsis** | |
| **Numerator** | Discharges among cases meeting the inclusion and exclusion rules for the denominator. With ICD-9-CM code for sepsis in any secondary diagnosis field. |
| **Denominator** | All elective* surgical discharges age 18 and older defined by specific DRGs or MS-DRGs and an ICD-9-CM code for an operating room procedure.  *Elective - SID Admission type # is recorded as elective (Admission Type = 3)  **Exclude cases:**   - with principal diagnosis of sepsis or secondary diagnosis present on admission. - with a principal diagnosis of infection. - with any code for immunocompromised state or cancer. - MDC 14 (pregnancy, childbirth, and puerperium). - with length of stay of less than 4 days. |
| **PSI#14 Postoperative Wound Dehiscence** | |
| **Numerator** | Discharges among cases meeting the inclusion and exclusion rules for the denominator with ICD-9-CM code for reclosure of postoperative disruption of abdominal wall procedure. |
| **Denominator** | All abdominopelvic surgical discharges age 18 and older.  **Exclude cases:**   - where a procedure for reclosure of postoperative disruption of abdominal wall occurs. before or on the same day as the first abdominopelvic surgery procedure. - where length of stay is less than 2 days. - with any diagnosis or procedure code for immunocompromised state. - MDC 14 (pregnancy, childbirth, and puerperium). |
| **PSI#18 Obstetric Trauma-Vaginal with instrument** | |
| **Numerator** | Discharges among cases meeting the inclusion and exclusion rules for the denominator. With ICD-9-CM codes for 3^rd^ and 4^th^ degree obstetric trauma in any diagnosis field. |
| **Denominator** | All vaginal delivery discharges with any procedure code for instrument-assisted delivery. |
| **PSI#19 Obstetric Trauma-Vaginal without instrument** | |
| **Numerator** | Discharges among cases meeting the inclusion and exclusion rules for the denominator with ICD-9-CM codes for 3^rd^ and 4^th^ degree obstetric trauma in any diagnosis field. |
| **Denominator** | All vaginal delivery discharge patients.  **Exclude cases:**   - with instrument-assisted delivery. |
| **PSI#20 Obstetric Trauma-Cesarean section** | |
| **Numerator** | Discharges among cases meeting the inclusion and exclusion rules for the denominator.with ICD-9-CM code for obstetric trauma in any diagnosis or procedure field. |
| **Denominator** | All Cesarean delivery discharges. |
